# A Novel Homozygous Mutation Destabilizes IKKβ and Leads to Human Combined Immunodeficiency

**DOI:** 10.3389/fimmu.2020.517544

**Published:** 2021-02-15

**Authors:** Tao Qin, Yanjun Jia, Yuhang Liu, Rongxin Dai, Lina Zhou, Satoshi Okada, Miyuki Tsumura, Hidenori Ohnishi, Zenichiro Kato, Hirokazu Kanegane, Xiulian Sun, Xiaodong Zhao

**Affiliations:** ^1^ Department of Infection, Children’s Hospital of Chongqing Medical University, Chongqing, China; ^2^ National Clinical Research Center for Child Health and Disorders, Chongqing, China; ^3^ Ministry of Education Key Laboratory of Child Development and Disorders, Chongqing, China; ^4^ China International Science and Technology Cooperation Base of Child Development and Critical Disorders, Chongqing, China; ^5^ Chongqing Key Laboratory of Child Infection and Immunity, Children’s Hospital of Chongqing Medical University, Chongqing, China; ^6^ Department of Rheumatism and Immunity, Children’s Hospital of Chongqing Medical University, Chongqing, China; ^7^ Department of Neurology, Children’s Hospital of Chongqing Medical University, Chongqing, China; ^8^ Chongqing Key Laboratory of Translational Medical Research in Cognitive Development and Learning and Memory Disorders, Children’s Hospital of Chongqing Medical University, Chongqing, China; ^9^ Department of Pediatrics, Hiroshima University Graduate School of Biomedical & Health Sciences, Hiroshima, Japan; ^10^ Department of Pediatrics, Gifu University Graduate School of Medicine, Gifu, Japan; ^11^ Structural Medicine, United Graduate School of Drug Discovery and Medical Information Sciences, Gifu University, Gifu, Japan; ^12^ Department of Child Health and Development, Graduate School of Medical and Dental Sciences, Tokyo Medical and Dental University, Tokyo, Japan; ^13^ Department of Brain Research Institute, Qilu Hospital of Shandong University, Shandong, China

**Keywords:** IKKβ, NF-κB, SCID, protein degradation, protein stability

## Abstract

Mutations in the *IKBKB* gene cause severe immunodeficiency, characterized clinically by persistent respiratory or gastrointestinal infections. Targeted gene panel sequencing revealed a novel homozygous missense mutation in the *IKBKB* gene of a patient with immune dysregulation and combined T and B cell functional defects. PBMCs from the patient, *Ikbkb* Y397H mice, and transfected cells were used to elucidate how the Y395H mutation triggers IKKβ deficiency and impairs immune function. Here, we found that cells from both the patient and *Ikbkb* Y397H mice lacked or showed decreased levels of IKKβ protein, along with impaired lymphocyte function. IKKα and IKKγ protein expression by human PBMCs harboring the Y395H mutation was normal, but degradation of IKKβ protein was accelerated. Binding of human NF-κB to DNA in patient PBMCs fell upon stimulation with TNF-α or LPS. Additionally, a structural model of Y395H revealed loss of the hydrogen bond with D389. These data suggest that *IKBKB* deficiency induces abnormal IKKβ protein degradation, leading to impaired NF-κB signaling and immune function. We postulate that the Y395H variant in the IKKβ protein lost the hydrogen bond with D389, thereby affecting interaction between Y395 and D389 and increasing protein instability.

## Introduction

Nuclear factor-κB (NF-κB) comprises a family of transcription factors crucial for cell survival, differentiation, and apoptosis (1). The NF-κB family includes p50, p52, RelA/p65, RelB, c-Rel, and the inhibitory subunits IκBα, IκBβ, and IκBϵ; IκBα is known to regulate NF-κB activation (2). In the absence of stimulatory signals the majority of inactive NF-κB is bound to IκBα and remains in the cell cytosol. The IκBα serine kinase (IKK) complex is activated by TNF, endotoxins, lymphotoxins, and viruses, resulting in dissociation of IκBα from NF-κB and subsequent nuclear translocation of the transcription factor (2). The predominant IKK complex contains two catalytic subunits, IKKα and IKKβ, and a regulatory subunit, IKKγ (also known as NEMO) (3–6). Activated IKKβ phosphorylates and dissociates IκBα from NF-κB by targeting serines 32 and 36 (7, 8). IKKα belongs to the serine/threonine protein kinase family, and NEMO acts as a scaffold protein essential for assembly of the IKK signalosome (9, 10).

*IKBKB* deficiency is a rare and special immunophenotype of severe immunodeficiency, which presents in neonates as persistent respiratory or gastrointestinal viral, bacterial, or fungal infections, often associated with protracted diarrhea and failure to thrive. Previous reports show that most *IKBKB* gene mutations are nonsense or duplication mutations, leading to lack of IKKβ protein expression and defective immune cell activation (11–15). The nonsense mutation (R286X) in the *IKBKB* gene results in an incomplete IKK complex and impaired IκBα activation (13). The studies mentioned above describe a marked severe immunodeficiency phenotype, with a mean age of mortality of 15 months, even when patients are treated with hematopoietic stem cell transplantation (HSCT).

Here, we describe a patient with early onset combined immunodeficiency who survived to adolescence. The patient harbors a homozygous missense mutation at amino acid 395 of the *IKBKB* gene, which accelerates degradation of the IKKβ protein and contributes to impaired lymphocyte function, disrupts NF-κB nuclear translocation, and causes an attenuated clinical phenotype.

## Materials and Methods

### Patient and Controls

The patient referred to the Children’s Hospital of Chongqing Medical University was from a consanguineous Chinese family. Whole blood samples were acquired from patient, the patient’s family members, and age-matched healthy volunteers. All provided informed consent. The patient was diagnosed by targeted gene panel sequencing, and the diagnosis was confirmed by Sanger sequencing. The average read-depth of sequencing was 309.58X and the percentage coverage of the exome at 20X was 91.7%. The capture reagent was a GenCap liquid capture Kit (MyGenostics, MD, USA) and sequencing was performed using an Illumina HiSeq 2500. The IKKβ protein of PBMCs (Peripheral blood mononuclear cells) was detected by flow cytometry analysis and western blotting of peripheral blood samples obtained prior to transplantation. The study was conducted in accordance with the Declaration of Helsinki and was approved by the ethics committee of Children’s Hospital of Chongqing Medical University.

### Immunologic Analysis

The patient underwent the following laboratory tests: white blood cell count, serum immunoglobulin levels, and lymphocyte subset counts. PBMCs were isolated from the patient and from healthy controls by Ficoll-Hypaque gradient centrifugation, and lymphocyte subsets were determined by flow cytometry (Canto, BD, USA) analysis, as previously described (16). Expression of T cell receptor excision DNA circles (TRECs) was determined by real-time polymerase chain reaction (17). Human CD4^+^CD25^+^FOXP3^+^ cells and the percentage of CD3^+^CD8^-^T cells that produced IL-4, IL-17, and IFN-γ were detected by flow cytometry, as described previously (18). Standard flow cytometry methods were used to stain for cell surface markers and for carboxyfluorescein diacetate succinimidyl ester dilution assays, which were used to analyze T cell proliferation after 3–4 days of culture with phytohemagglutinin (PHA) and phytolacca americana (PWM) (19). CDR3 spectratyping analysis of TCR Vβ was performed to define each subfamily as normal or skewed (20). Expression of CD107α by natural killer (NK) cells was detected after stimulating K562 cells for 4 h with PBMCs at a ratio of 1:5. Production of IL-12 and IFN-γ of PBMCs was measured in response to stimulation with BCG alone, and in response to BCG plus exogenous recombinant IFN-γ or IL-12 (21).

### Detection of the IKK Complex

Degradation of human IKKβ protein was detected by flow cytometry. Briefly, freshly isolated PBMCs were stimulated for the indicated times with CHX (cycloheximide, 2 μg/mL; Cat.C7698, Sigma, USA) and IKKβ expression examined. Stimulated PBMCs were fixed, permeabilized (in Cytofix/Cytoperm, BD, USA), stained with a rabbit anti-IKKβ monoclonal antibody (1:100 dilution, ab32135, Abcam, UK) or isotype IgG (1:100 dilution, ab172730, Abcam, UK) for 20 min, and incubated with phycoerythrin (PE)-conjugated goat anti-rabbit IgG H&L (1:50–1:100 dilution, ab97070, Abcam, UK) for 20 min. Finally, cells were analyzed using a FACSCanto flow cytometer (BD, USA). To detect human IKKα and IKKγ, PBMCs were isolated from freshly drawn heparinized blood by Ficoll density gradient centrifugation, resuspended (2×10^6^ cells/mL) in medium (RPMI 1640 containing 10% fetal calf serum, 200 μg/mL penicillin, 200 U/mL streptomycin, and 4 mM L-glutamine [all from INVITROGEN, CA, USA]), and stimulated for 1, 2, or 4 h with LPS (lipopolysaccharide, 10 μg/mL; Cat.L4516 B4; SIGMA-ALDRICH, St. Louis, MI, USA) in 48-well flat-bottom plates. Before flow cytometry analysis, cells were fixed, permeabilized, and stained with rabbit anti-IKKα, an anti-IKKγ monoclonal antibody (1:100 dilution, ab32041 or ab137363, Abcam, UK), or a rabbit IgG monoclonal antibody (1:100 dilution, ab172730, Abcam, UK), followed by PE-conjugated goat anti-rabbit IgG H&L (1:50–1:100 dilution, ab97070 Abcam, UK). Samples were analyzed on a FACSCanto flow cytometer (BD, USA).

Expression vectors harboring pCMV3-C-His-IKKβ^WT^, IKKβ^Y395H^, and IKKβ^Y395E^ (We generated the Y395E mutant to have a different amino acid change at the 395 residue) were transfected separately into HEK293 cells (in 60-mm cell culture plates) using Lipofectamine 2000 (Invitrogen, Waltham, MA, USA). After 48 h, cells were harvested and pre-stimulated for the indicated times with CHX (2 μg/ml; Cat.C7698, Sigma-Aldrich, USA) prior to lysis and we used the His tag antibodies to detect IKKβ protein by western blotting and ELISA at 0, 12, 24, 48 and 72 h.

### Electrophoretic Mobility Shift Assay

PBMCs were stimulated for 4 h with LPS or TNF-α (10 ng/ml), lysed to harvest nucleoproteins, and NF-κB binding to DNA detected in an electrophoretic mobility shift assay (EMSA). Expression vectors harboring pCMV3-C-His-IKKβ^WT^, IKKβ^Y395H^, and IKKβ^Y395E^ were transfected separately into HEK293 cells. After 48 h, cells were stimulated for 24 h with CHX, lysed to harvest nucleoproteins, and NF-κB binding to DNA detected in EMSA with Lightshift Chemiluminescent EMSA Kit (Cat.20148, Thermo Scientific). To ensure comparable levels of expression of the three kinds of cells, we used the same vector and the cell density, state and transfection system were consistent during cell transfection. The protein concentrations were detected and adjusted to make the sample volumes consistent.

### Protein Structure

Structural data for the human IKKβ protein (PDB code: 4KIK) were used (22). The structure of the Y395H mutant was built using MOE software (Molecular Operating Environment 2013.08; Chemical Computing Group Inc., www.chemcomp.com) and structural figures prepared using PyMOL 2.2.

### Mice

C57BL/6J-*IKBKB^tm1(Y397H)Smoc^* mice were generated by the Shanghai Biomodel Organism Science & Technology Development Company using the CRISPR/Cas9 system. Mice were maintained in horizontal laminar flow cabinets in a specific pathogen-free facility and provided with sterile food and water *ad libitum*. All animal experiments were conducted in accordance with the institutional guidelines of Chongqing Medical University. Expression of IKKα, IKKβ, and IKKγ in thymus and spleen cells from WT and Y397H mice was detected by western blotting with rabbit monoclonal antibodies specific for IKKα (1:1,000 dilution, ab32041, Abcam, UK), IKKβ (1:1,000 dilution, ab124957, Abcam, UK), or IKKγ (1:1,000 dilution, ab178872, Abcam, UK). The lymphocyte subsets within cell populations from the thymus and spleen of WT and Y397H mice were analyzed by flow cytometry.

### Statistical Analyses

All statistical analyses were performed using SPSS version 22.0 software. Data are presented as the mean ± standard deviation. Groups of mice were compared using unpaired t-tests. *P <*0.05 was considered statistically significant.

## Results

### Clinical Characteristics

The patient presented with clinical features characteristic of *IKBKB*-deficient individuals. The patient became ill at 2 months-of-age, suffering repeated respiratory and urinary tract infections (5–6 times per year), constant otitis media, and severe chronic diarrhea coupled with marked growth retardation (height, 142 cm at 17 years-of-age; significantly less than three percent, weight, 26 kg at 17 years-of-age; significantly less than three percent). He had a history of abscess formation at the BCG vaccine site without regional lymphadenopathy. His parents are first cousins. Laboratory tests revealed hypogammaglobulinemia [extremely low IgG (1.88–2.75 g/L; normal range, 8.27–14.17g/L), IgM (0.114–0.159 g/L; normal range, 1.22–2.56g/L), and IgA (<0.067 g/L; normal range, 0.86–1.92g/L) levels], which did not return to normal after intravenous immune globulin. We speculate that the reason for the low immune globulin level was irregular intravenous immune globulin replacement therapy. However, lymphocyte counts (5.81–10.41×10^9^ cells/L) were normal. White blood cell counts were elevated consistently (13.2–18.5×10^9^ cells/L) and there was a history of candida albicans infection upon fecal testing. The TRECs level was low (40.03/10^5^ cells; normal range, 150–360/10^5^ cells). Gastroscopy and colonoscopy revealed chronic inflammation. His condition was stabilized by administration of intravenous immunoglobulin (400–500 mg/kg per month since the first visit to our hospital) prior to HSCT. The patient received HSCT from his HLA-matched older sister after pretreatment for 7 days with fludarabine, busulfan, and anti-thymocyte globulin. HSCT was successful and STR-PCR (short tandem repeat-polymerase chain reaction) was 100% which was tested 27 days after the patient received the transplantation. He recovered and regained normal weight, suffered only mild graft-versus-host disease symptoms (gingival hyperplasia and itchy skin), and had no further diarrhea and respiratory tract infections.

### A Novel *IKBKB* Gene Mutation

**Figure 1:**
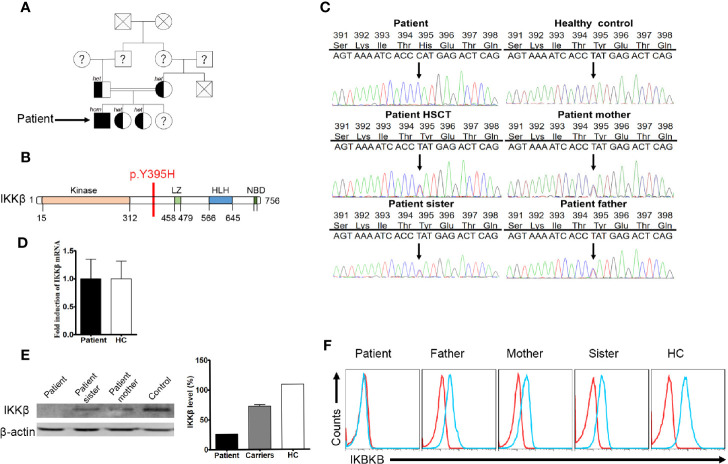
A missense mutation detected by targeted gene panel sequencing and Sanger sequencing. **(A)** The family of the immunodeficient child. Squares denote male family members, circles denote female members. Solid symbols denote family members who were homozygous for the *IKBKB* mutation, half-solid symbols denote members who were heterozygous for the mutation; family members not tested are indicated by question marks and family members that have passed away are indicated by crosses. **(B)** Predicted impairment of the α-helical scaffold dimerization domain encompassing the leucine-zipper, helix-loop-helix domains, and the nuclear factor-κB (NF-κB) essential modulator (NEMO)-binding domain (NBD) of IκB kinase 2. **(C)** *IKBKB* sequence analysis of DNA from the patient, healthy controls, and family members. The sequence harbors a homozygous point mutation (T→C) at position 1183 in exon 12, which results in substitution of a histidine for tyrosine at position 395 (Y395H). **(D)** Expression of *IKBKB* mRNA in patient and healthy controls. N≥3. **(E)** Western blot analysis of IKKβ protein expression in peripheral blood mononuclear cells (PBMCs) from the patient, his family members, and a healthy control. N≥3. Quantification revealed that the patient had low IKKβ levels. **(F)** Flow cytometry analysis of IKKβ protein expression by lymphocytes isolated from the peripheral blood of the patient, his family members, and a healthy control. N≥3.

We assumed autosomal recessive inheritance of a founder allele and so conducted sequence analysis of both the patient and healthy siblings (**Figure 1A**). Patient samples were subjected to targeted gene panel sequencing to determine genetic alterations (**Supplementary Table 1**). A mutation in the *IKBKB* gene was located on chromosome 8 (**Figure 1B**). SOAPsnp software was used to detect SNPs by comparing healthy volunteer and patient files with CCDS, dbSNP, and the Human Genome Database. Protein functions were predicted using SIFT. Insertions and deletions (indels) were detected using the Burrows–Wheeler Aligner and GATK software, and were defined using CCDS, dbSNP, and the Human Genome Database. Candidate mutations identified through sequencing were further tested by Sanger sequencing. The patient harbored a homozygous point mutation (T≥C) at position 1,183 in exon 12 of the *IKBKB* gene, which resulted in substitution of histidine with tyrosine at position 395 (Y395H). The mutation has not been identified in the 1,000 genome, gnomAD_genome_ALL, esp6500siv2_all, ExAC_ALL, and Inhouse databases. His family members also carried this mutation. After HSCT, the patient harbored a heterozygous mutation in peripheral blood cells (**Figure 1C**). The mutation found in the patient had not yet been reported in SNP databases and was not observed in 1,000 control subjects. Sanger sequencing of genomic DNA and cDNA using samples from the patient and his family members confirmed the point mutation. Expression of *IKBKB* mRNA was normal (compared with that in healthy controls) (**Figure 1D**). Western blot analysis revealed that the patient lacked expression of the IKKβ protein (**Figure 1E**). Family members showed partial expression of the IKKβ protein (levels were lower than those in healthy controls but higher than those in the patient). The same results were obtained by flow cytometry (**Figure 1F**).

### Impaired Immunologic Function

**Figure 2:**
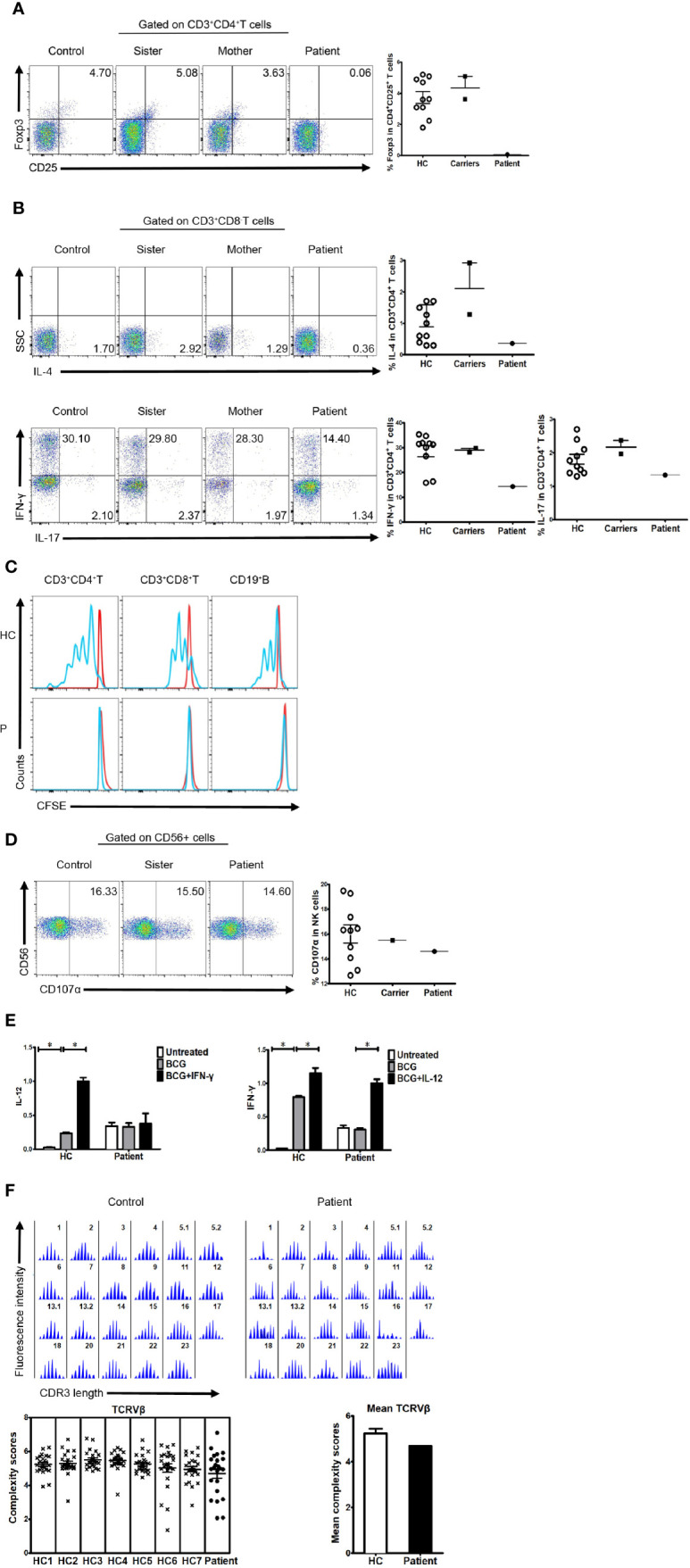
Immunologic function in the patient, his family members, and healthy controls. **(A)** Percentage of FOXP3-expressing cells within the CD4^+^CD25^+^ T cell population of healthy controls, family members, and a patient (3.732 ± 1.206%, n=10 *vs.* 4.355 ± 1.025%; n=2 *vs.* 0.063; n=1). **(B)** Percentage of cytokine-producing cells within the CD3^+^CD8^-^T cell population of healthy controls, family members, and the patient. IL-4 (1.243 ± 1.149%, n=11 *vs.* 2.105 ± 1.153%, n=2 *vs.* 0.365, n=1, respectively); IL-17 (1.807 ± 0.467%, n=10 *vs.* 2.170 ± 0.282, n=2 *vs.* 1.340, n=1, respectively); IFN-γ (28.550 ± 6.960%, n=10 *vs.* 29.050 ± 1.061%, n=2 *vs.* 14.400, n=1, respectively). **(C)** Impaired lymphocyte proliferation in the patient. N≥3. **(D)** Percentage of CD107α cells within the CD56^+^ NK cell population of healthy controls, family members, and the patient (16.000 ± 2.344%, n=10 *vs.* 15.500, n=1 *vs.* 14.600, n=1, respectively). **(E)** Real-time polymerase chain reaction (PCR) analysis of expression of IL-12 and IFN-γ in the patient’s PBMCs stimulated with BCG plus IFN-γ or IL-12. N≥3. **(F)** TCRVβ diversity in the patient and healthy controls. N≥3.

Like patients reported before, our patient suffered impaired immunologic function. The percentage and number of CD27^+^ memory B cells in the patient were lower than the normal reference ranges (**Table 1**). In addition, the percentage of CD4^+^CD25^+^FOXP3^+^ T cells was much lower than that in healthy controls (**Figure 2A**). The percentage of CD3^+^CD8^-^ T cells producing IL-4, IL-17, and IFN-γ was also lower than that in healthy controls (**Figure 2B**). Finally, proliferation for T and B lymphocytes was impaired (**Figure 2C**).

**Table 1:** Immunologic characteristics of lymphocytes.

Cell population	Patient counts (% of each cell type)	Patient (cells per cubic millimeter)	Normal range (cells per cubic millimeter)
T cell			
CD3^+^	76.37 (Lymph.)	5,865.2 ↑	1,019.3–2,688.4
CD3^+^CD4^+^	36.00 (CD3^+^)	2,111.2 ↑	306.3–1,256.9
CD3^+^CD4^+^ CD27^+^CD45RA^-^ (central memory)	75.30 (CD3^+^CD4^+^)	1,589.8 ↑	132.6–466.2
CD3^+^CD4^+^ CD27^+^CD45RA^+^ (naïve)	22.30 (CD3^+^CD4^+^)	470.8	158.0–924.0
CD3^+^CD4^+^ CD27^-^CD45RA^-^ (effector memory)	2.40 (CD3^+^CD4^+^)	50.7	18.7–189.5
CD3^+^CD4^+^ CD27^-^CD45RA^+^ (TEMRA)	0 (CD3^+^CD4^+^)	0	0–25.5
CD3^+^CD8^+^	58.95 (CD3^+^)	3457.5 ↑	359.2–1,083.4
CD3^+^CD8^+^CD27^+^CD45RA^-^ (central memory)	27.80 (CD3^+^CD8^+^)	961.2 ↑	60.8-283.3
CD3^+^CD8^+^CD27^+^CD45RA^+^ (naïve)	39.10 (CD3^+^CD8^+^)	1351.9 ↑	189.5–628.7
CD3^+^CD8^+^ CD27^-^CD45RA^-^ (effector memory)	9.10 (CD3^+^CD8^+^)	314.6 ↑	5.7–213.3
CD3^+^CD8^+^CD27^-^CD45RA^+^ (TEMRA)	24.00 (CD3^+^CD8^+^)	829.8 ↑	10.3–374.6
CD3^+^ α/β^+^	1.47 (CD3^+^)	86.1 ↑	8.9–61.6
CD3^+^ γ/δ^+^	8.20 (CD3^+^)	480.9 ↑	64.7-479.7
CD3^+^ γ/δ2^+^	3.27 (CD3^+^)	192.0	30.5–233.5
B cells			
CD19^+^	7.1 (Lymph.)	545.3 ↑	143.1–534.2
CD19^+^CD24^+^CD38^+^ (transitional)	4.80 (CD19^+^)	26.2	1.4–37.8
CD19^+^CD24^-^CD38^+^ (Plasma blasts)	0.40 (CD19^+^)	2.2	1.9–36.2
CD19^+^CD27^+^IgD^-^ (memory)	0.70 (CD19^+^)	3.8 ↓	18.7–103.9
CD19^+^CD27^-^IgD^+^ (naive)	94.00 (CD19^+^)	512.6 ↑	81.2–400.9
NK cells			
CD56^+^16^+^	15.42 (Lymph.)	1184.3 ↑	121.5–1010.7

Expression of CD107α in NK cells was normal (**Figure 2D**). Furthermore, cells from the patient showed reduced production of IL-12 in response to IFN-γ (**Figure 2E**), suggesting that the IFN-γ/IL-12 axis was impaired. To further dissect rearrangement of the T cell receptor, we examined the length of the TCR CDR3 in total peripheral T cells from the patient and healthy control subjects. The majority of Vβ subfamilies in T cells from control subjects exhibited six or more peaks with a Gaussian distribution, indicating a polyclonal Vβ repertoire. By contrast, a number of Vβ subfamilies in T cells from the patient displayed skewed spectratyping profiles, indicating monoclonal or oligoclonal proliferation of T cells. However, there was no significant difference in the mean complexity scores between the patient and healthy controls (**Figure 2F**).

### Altered Structure of the Human IKKβ Y395H Protein

**Figure 3:**
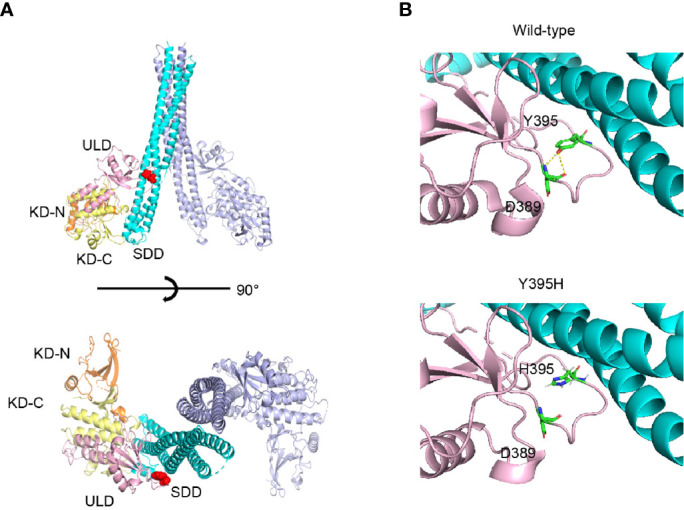
Protein structure of human IKKβ. **(A)** The location of Y395H within the human IKKβ protein is indicated by the red sphere. **(B)** Structural comparison of wild-type and the Y395H mutant. The hydrogen bond (yellow line) between the -OH of the Y395 side chain and the -NH2 of the D389 main chain was lost.

The mutated residue, Y395, is located in the ubiquitin-like domain of human IKKβ. This residue forms a part of the loop that interacts with the neighbor scaffold/dimerization domain (**Figure 3A**). As shown in **Figure 3B**, the model structure of Y395H shows loss of the hydrogen bond with D389, suggesting that the loop conformation is unstable. Therefore, Y395H might affect the stability of the protein by disrupting the interaction between Y395 and D389.

### Susceptibility of Mutant IKKβ Protein to Degradation and Impaired NF-κB Signaling

**Figure 4:**
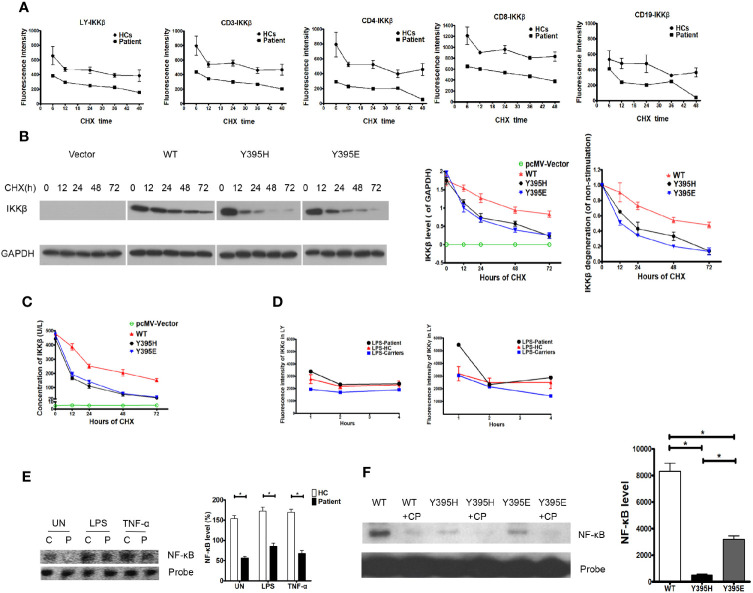
Degradation of the IKKβ protein in the patient and in transfected cells. **(A)** IKKβ was degraded faster in patient PBMCs than in those of healthy controls upon incubation with cycloheximide (CHX). N≥3 for healthy controls and N=1 for patient. **(B)** IKKβ was degraded faster in IKKβ^Y395H^ HEK293 cells than in IKKβ^WT^ HEK293 cells in the presence of CHX by western blotting. N≥3. **(C)** IKKβ was degraded faster in IKKβ^Y395H^ HEK293 cells than in IKKβ^WT^ HEK293 cells in the presence of CHX by ELISA. N≥3. **(D)** Expression of IKKα and IKKγ was normal in patient PBMCs stimulated by LPS. N≥3 for healthy controls and N=1 for patient. **(E)** NF-κB binding to DNA in nucleoprotein of patient PBMCs, as measured in an electrophoretic mobility shift assay. N=3, *P<0.005. Quantification revealed that NF-κB binding to DNA in patient cells was lower than in the control group, the TNF-α-treated group, and the LPS-treated group. **(F)** NF-κB binding to DNA in nucleoprotein of IKKβ^Y395H^ HEK293 cells, IKKβ^Y395E^ HEK293 cells and IKKβ^WT^ HEK293 cells, as measured in an electrophoretic mobility shift assay. CP is for cold probe. N=3, *P<0.005.

Data obtained thus far suggest that the patient harbored a missense mutation in the *IKBKB* gene, although mRNA expression was normal. However, expression of the IKKβ protein was impaired, and the loop conformation of IKKβ *Y395H* was unstable, indicating that the mutation had the potential to trigger degradation of the corresponding protein. Thus, we examined dynamic expression of the IKKβ protein in the presence of CHX and found that it was lower in the patient’s cells than in those from healthy controls. Furthermore, the IKKβ protein in the patient’s cells looked likely to be more vulnerable to degradation than that in healthy control cells, regardless of whether we tested whole lymphocyte preparations or lymphocyte subsets (**Figure 4A**). To confirm that IKKβ protein harboring the *IKBKB* Y395H mutation was more vulnerable to degradation, the expression vectors harboring IKKβ^WT^, IKKβ^Y395H^, and IKKβ^Y395E^ were transfected into HEK293 cells. And the IKKβ protein was degraded faster in IKKβ^Y395H^ HEK293 cells and IKKβ^Y395E^ HEK293 cells by western blotting (**Figure 4B**) and ELISA (**Figure 4C**).

Expression of IKKα and IKKγ in the patient’s cells in response to LPS was similar to that in cells from healthy controls and family members carrying the gene mutation (**Figure 4D**). To elucidate whether NF-κB signaling was affected by the IKKβ mutation, we examined NF-κB binding to DNA in electrophoretic mobility shift assay. We found that NF-κB binding to DNA in patient cells decreased under untreated conditions (153.70 ± 7.51% *vs.* 56.67 ± 3.84%, P=0.0003), and that NF-κB binding to DNA in patient cells stimulated with TNF-α was slightly inhibited (169.00 ± 8.08% *vs.* 67.67 ± 7.84%, P=0.0008). Similar results were observed upon LPS simulation (172.70 ± 9.77% *vs.* 85.00 ± 7.94%, P=0.0022) (**Figure 4E**). And we also found that NF-κB binding to DNA decreased in IKKβ^Y395H^ HEK293 cells (495.7 ± 76.67) and IKKβ^Y395E^ HEK293 cells (3199.00 ± 261.30) than in IKKβ^WT^ HEK293 cells (8315.00 ± 617.40) (**Figure 4F**).

### *IKBKB* Y397H Mice

**Figure 5:**
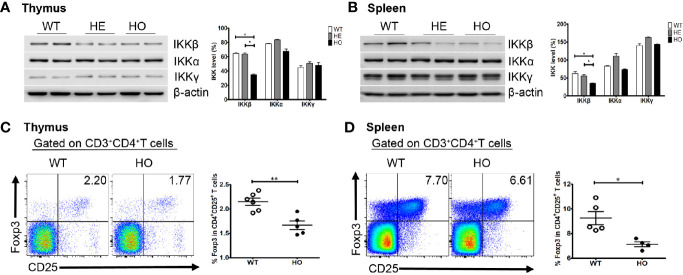
Expression of the IKK complex and the percentage of Treg cells in mice. **(A)** Expression of the IKK complex in thymus cells from *Ikbkb* Y397H mice, heterozygous mice, and WT mice. Quantification revealed that IKKβ levels lower in in *Ikbkb* Y397H mice than in heterozygous mice and WT mice. N≥3. **(B)** Expression of the IKK complex in spleen cells from *Ikbkb* Y397H mice, heterozygous mice, and WT mice. Quantification revealed that IKKβ levels were lower in *Ikbkb* Y397H mice than in heterozygous mice and WT mice. N≥3. **(C)** Percentage of CD4^+^CD25^+^FOXP3^+^ cells within the thymus cell population of *Ikbkb* Y397H and WT mice (2.150 ± 0.179%, n=6 *vs.* 1.668 ± 0.196%, respectively; n=5, P=0.0021). **(D)** Percentage of CD4^+^CD25^+^FOXP3^+^ cells within the spleen cell population of *Ikbkb* Y397H and WT mice (9.248 ± 1.182%, n=5 *vs.*7.133 ± 0.416%, n=4, respectively; P=0.0118). Numbers in the indicated quadrants represent the percentage of cells in that gate. Data are representative of 4–6 independent experiments.*P< 0.05, and **P<0.01, unpaired t-test.

We generated a mouse model using a CRISPR/Cas9 gene targeting strategy in C57BL/6J mouse embryos to introduce a homozygous Y397H mutation into the *Ikbkb* gene (**Supplementary Figure 1**), which is equivalent to the *IKBKB* Y395H mutation in the patient. Homozygous mutant mice (hereafter referred to as *Ikbkb* HO mice) were fertile and born at normal Mendelian ratios. The mice showed no obvious anatomical or behavioral abnormalities. Expression of *Ikbkb* mRNA in the thymus and spleen of mutant mice was comparable with that in wild-type mice, as were the levels of other IKK members (IKKα and IKKγ). However, we found that expression of IKKβ protein in the thymus or spleen of heterozygous (HE) and homozygous (HO) mice was lower than that in wild-type mice, while expression of IKKα and IKKγ protein was not significantly different between WT and HO mice (**Figures 5A, B** **)**. There were no obvious changes in the percentage and number of T and B cell subsets in mutant mice (**Supplementary Figure 2**), except for a slight decrease in the percentage of regulatory T cells (Tregs) in the thymus and spleen of mutant mice compared with WT mice (**Figures 5C, D** **)**.

## Discussion

Here, we identified a patient who presented with recurrent respiratory tract infections, severe and continual diarrhea, urinary tract infection, tympanitis, and growth retardation. The patient harbors an autosomal recessive *IKBKB* mutation resulting in loss of IKKβ protein expression; his parents and older sisters harbor a heterozygous mutation and show reduced IKKβ protein expression; however, they are healthy. The patient had hypogammaglobulinemia but normal T cell and B cell counts. The noticeable expansion of CD8-positive T cells may be related to repeated virus infection. However, we do not have any etiological evidence. Analysis of PBMCs isolated from the patient revealed an extremely low percentage of memory B cells and Treg cells, impaired lymphocyte proliferation, and reduced expression of IL-17, IFN-γ, and IL-4. Immunologic and functional investigations revealed that Y395 of IKKβ is necessary for differentiation of Tregs, which was confirmed in *Ikbkb* Y397H mice and in other conditional mouse models of IKKβ deficiency (23, 24). And reduced production of IL-12 in response to IFN-γ in patient suggested that impaired IFN-γ/IL-12 axis and defective myeloid cell function.

Previous studies report point and frame shift mutations that result in a premature stop codon, which has the potential to induce nonsense-mediated decay of the corresponding mRNAs, leading to severely truncated IKKβ protein or impaired IKKβ protein expression and function (11–13). However, the patient harbored a missense mutation, resulting in normal RNA expression but loss of IKKβ protein expression; this indicates that tyrosine at position 395 of *IKBKB* is important for function. A structural model of the IKKβ H395 protein revealed that the hydrogen bond with D389 was lost, suggesting that the loop conformation becomes unstable, thereby affecting interaction between these two domains. Furthermore, we found that expression of the IKKβ protein was degraded more rapidly in both patient PBMCs and in IKKβ^Y395H^ HEK293 cells in the presence of CHX. It is possible that substitution of histidine for tyrosine at position 395 may alter the protein’s structure and, therefore, stability. In addition, we examined expression of IKKα and IKKγ proteins, and found that not only did the patient have normal IKKα and IKKγ protein expression, but also that the mutation had no effect on expression of other IKK subunits. The vast majority of studies suggest that IKKβ undergoes phosphorylation on residues serine and tyrosine (25–27) (serine residues 177/181 and tyrosines 188/199), which are crucial for IKKβ activation. Further studies are needed to ascertain whether phosphorylation of the tyrosine residue at position 395 is required for proper activation of IKKβ.

IKKβ activates the canonical NF-κB pathway. Activated IKKβ phosphorylates IκBα to dissociate it from NF-κB, resulting in translocation of NF-κB to the nucleus and binding to its cognate transcription factor. Therefore, we examined NF-κB binding to DNA we found a marked reduction in *IKBKB* Y395H in the presence/absence of stimulation. This suggests that the Y395H mutation might affect the interaction between NF-κB and DNA in the nucleus, leading to immune deficiency or dysregulation. However, the effects are less serious than those of nonsense mutations and frame shift mutations.

The findings reported herein show that a homozygous missense mutation in *IKBKB* gene manifests with a milder clinical phenotype than the homozygous nonsense or frame shift mutations reported previously (11–13). Rare mutations in *IKBKB* have been reported, which lead to absence of protein expression and confer severe early onset immunodeficiency with hypogammaglobulinemia or agammaglobulinemia, low memory B cell numbers, impaired lymphocyte proliferation, NF-κB signaling, lymphocyte differentiation and activation, and deficiency of Treg cells and γδ T cells (11–13). Here, we found that both the patient and *Ikbkb* Y397H mice lacked or had reduced numbers of Treg cells. Some studies show that FOXP3 expression is regulated by canonical NF-κB signaling (28, 29). Also, a previous study of canonical NF-κB signaling defects shows a deficiency of both conventional T cells and Treg cells (30).

Taken together, these data describe a potentially novel mechanism for IKKβ activation. Tyrosine 395 appears to be crucial for this process because the mutation inhibits NF-κB binding to DNA. We will continue to explore tyrosine 395 phosphorylation and try to identify potential specific phosphokinases and phosphatases. In addition, the result suggests that IKKβ activation can be regulated, and that tyrosine 395 is a potential target for therapeutic interventions to treat infections and cancer.

## Data Availability Statement

The raw data supporting the conclusions of this article will be made available by the authors, without undue reservation, to any qualified researcher.

## Ethics Statement

The studies involving human participants were reviewed and approved by Medical research ethics committee of Chongqing Medical University. Written informed consent to participate in this study was provided by the participants’ legal guardian/next of kin. The animal study was reviewed and approved by Medical research ethics committee of Chongqing Medical University.

## Author Contributions

TQ, YJ, and YL designed and performed experiments, and analyzed the data. TQ wrote the first draft of the manuscript. RD, LZ, SO, MT, HO, ZK, and XS performed the experiments. HK contributed to scientific discussion, data interpretation, and revision of the manuscript. XZ designed the research, supervised the study, and revised the manuscript. All authors contributed to the article and approved the submitted version.

## Funding

The study was funded by the Public Welfare Scientific Research Project of China (201402012) and by the National Natural Science Foundation of China (81901611).

## Conflict of Interest

The authors declare that the research was conducted in the absence of any commercial or financial relationships that could be construed as a potential conflict of interest.

## Supplementary Material

Supplementary Figure 1
*IKBKB* sequence analysis of DNA from the *Ikbkb* Y397H mice.

Supplementary Figure 2 Expression of the T cell subsets and B cell subsets in mice. **(A)** Expression of CD4^+^ T cells, CD8^+^ T cells, CD4^+^CD8^+^ T cells, CD4^-^CD8^-^ T cells population of *Ikbkb* Y397H and WT mice. N≥3. **(B)** Expression of naïve T cells, central memory T cells and effector memory T cells in CD4^+^ T cells and CD8^+^ T cells population of *Ikbkb* Y397H and WT mice. N≥3. **(C)** The proportions of B cell in bone marrow and spleen from mutant mice and wide type mice. N≥3. MZ/MZP is for marginal zone/marginal zone precursor. T1/T2 is for transitional B cells. FM is for follicular B-cells. IS is for Class switching cell subsets.

## Abbreviations

CHX, Cycloheximide; HSCT, Hematopoietic stem cell transplantation; LPS, Lipopolysaccharide; NF-κB, Nuclear factor-κB; PBMCs, Peripheral blood mononuclear cells; SCID, Severe combined immunodeficiency.
