# Supplementary figures and images for: A Novel Homozygous Mutation Destabilizes IKKβ and Leads to Human Combined Immunodeficiency
Source: Front Immunol. 2021 Feb 15;11:517544. doi: 10.3389/fimmu.2020.517544 (PMC7917045; doi:10.3389/fimmu.2020.517544)

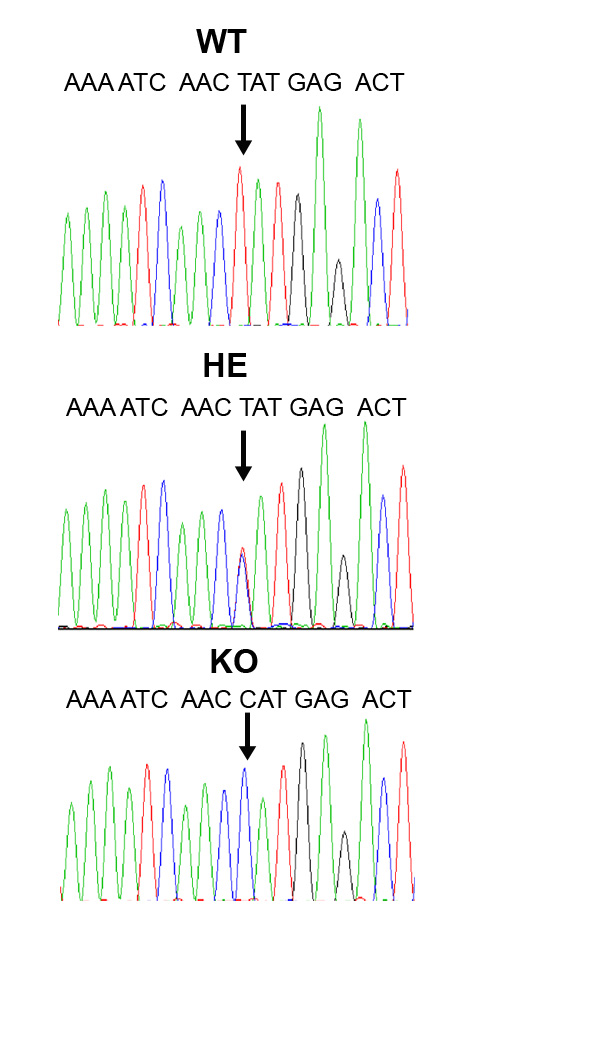

Supplement: Supplementary Figure 1 — IKBKB sequence analysis of DNA from the Ikbkb Y397H mice. [file Image_1.jpeg]

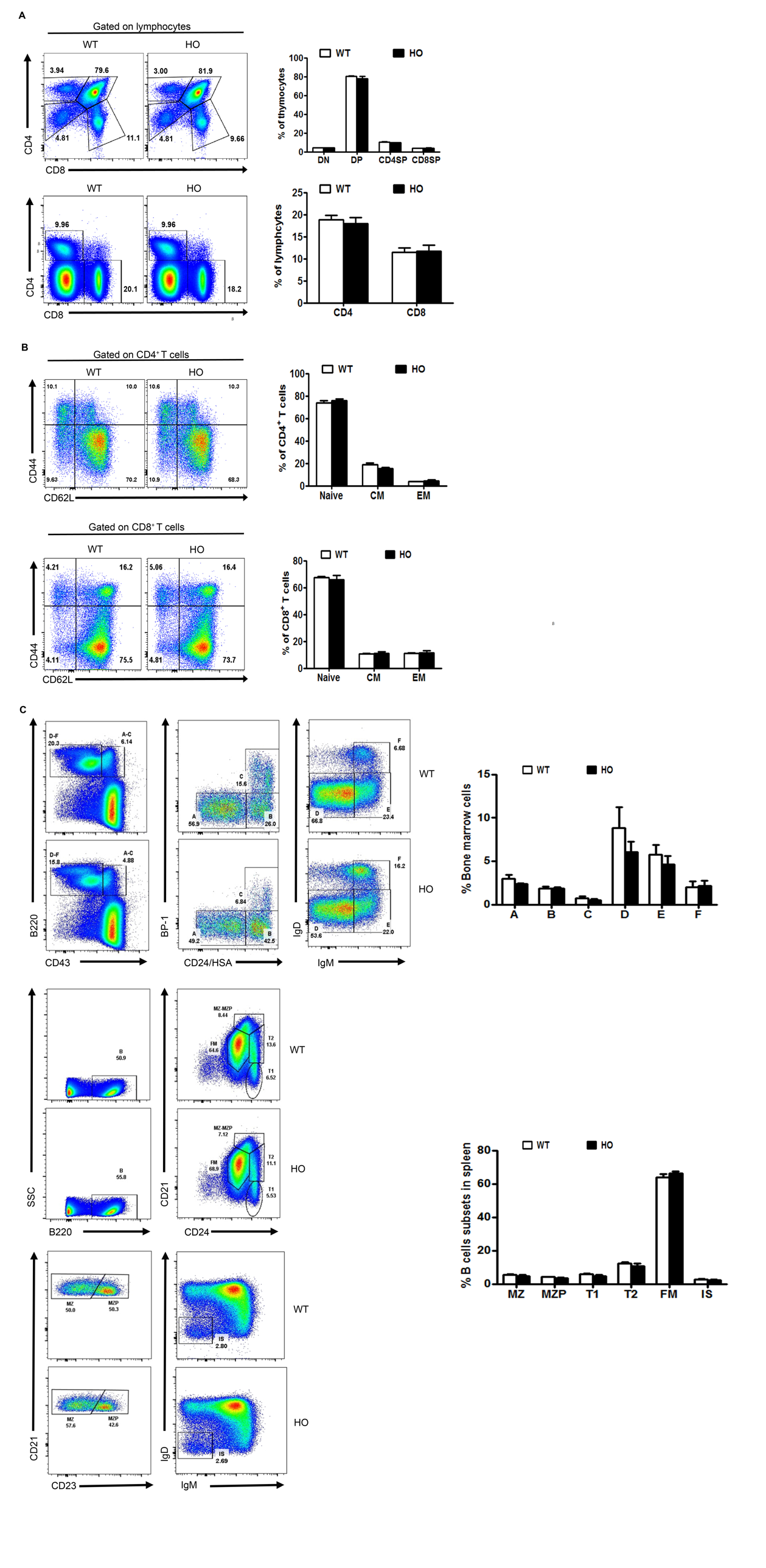

Supplement: Supplementary Figure 2 — Expression of the T cell subsets and B cell subsets in mice. (A) Expression of CD4+ T cells, CD8+ T cells, CD4+CD8+ T cells, CD4-CD8- T cells population of Ikbkb Y397H and WT mice. N≥3. (B) Expression of naïve T cells, central memory T cells and effector memory T cells in CD4+ T cells and CD8+ T cells population of Ikbkb Y397H and WT mice. N≥3. (C) The proportions of B cell in bone marrow and spleen from mutant mice and wide type mice. N≥3. MZ/MZP is for marginal zone/marginal zone precursor. T1/T2 is for transitional B cells. FM is for follicular B-cells. IS is for Class switching cell subsets. [file Image_2.jpeg]

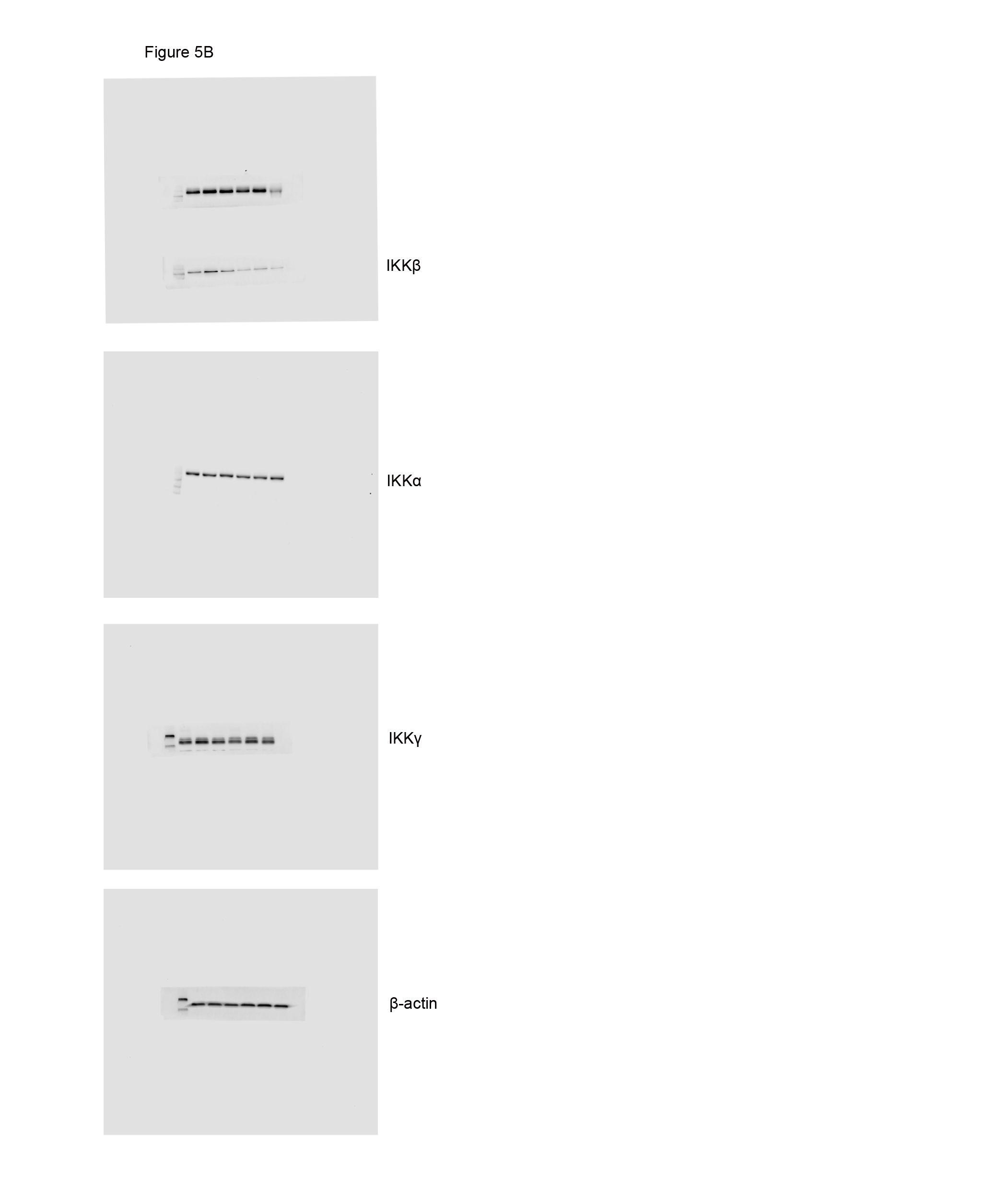

Supplement: Supplementary file 3 [file DataSheet_1.zip › Data sheet/image files of blots/Figure 5B.tif]

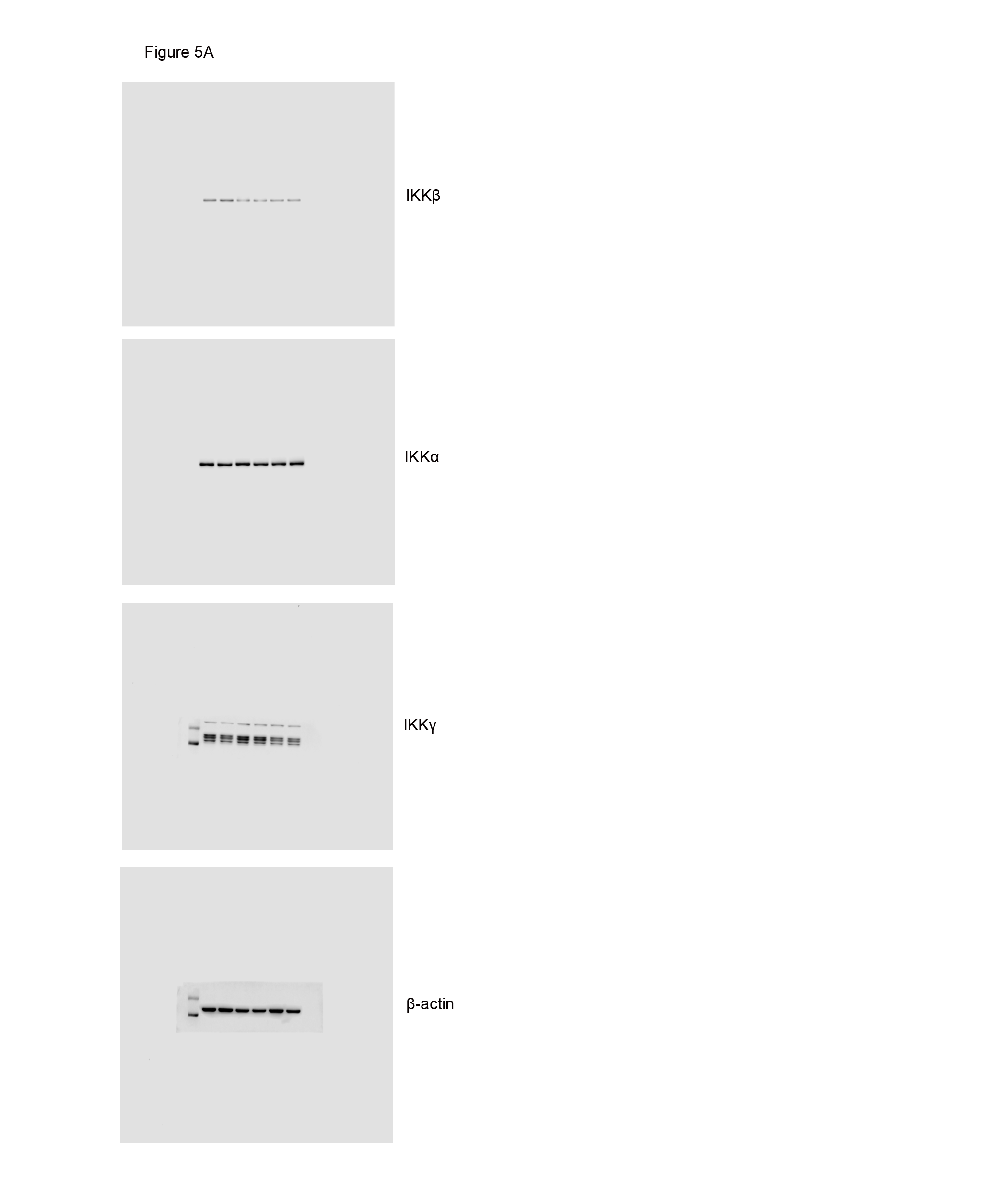

Supplement: Supplementary file 3 [file DataSheet_1.zip › Data sheet/image files of blots/Figure 5A.tif]

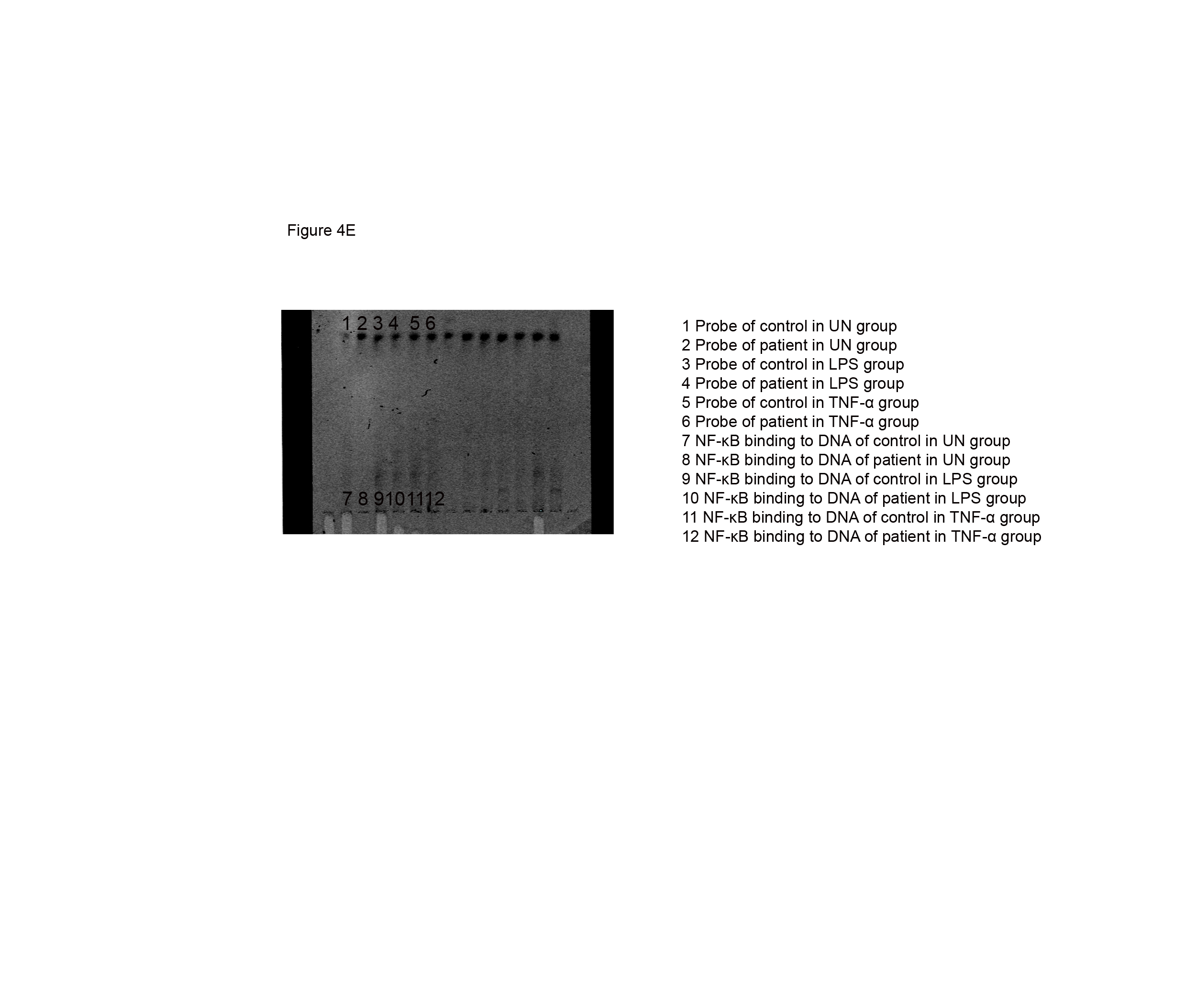

Supplement: Supplementary file 3 [file DataSheet_1.zip › Data sheet/image files of blots/Figure 4E.tif]

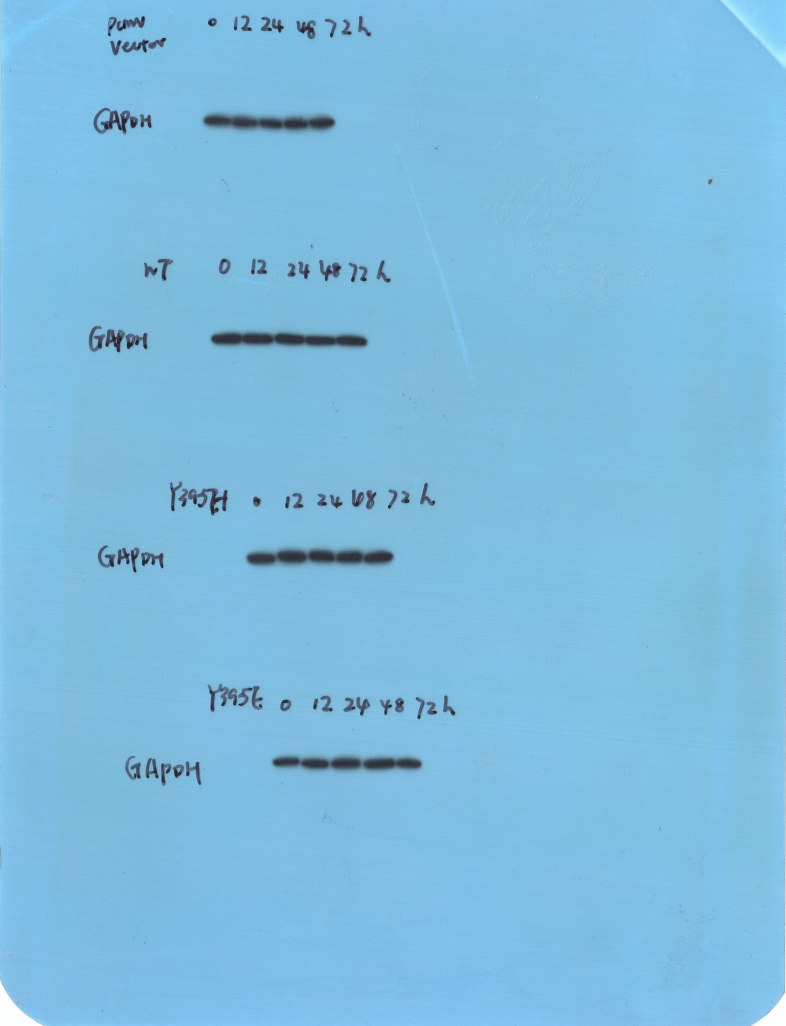

Supplement: Supplementary file 3 [file DataSheet_1.zip › Data sheet/image files of blots/Figure 4B-GAPDH.jpg]

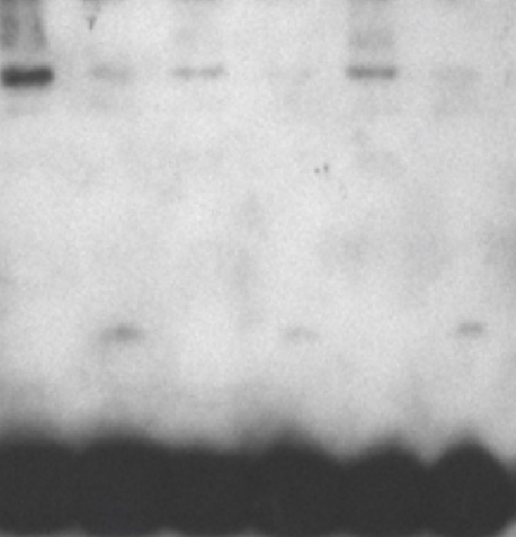

Supplement: Supplementary file 3 [file DataSheet_1.zip › Data sheet/image files of blots/Figure 4F.jpg]

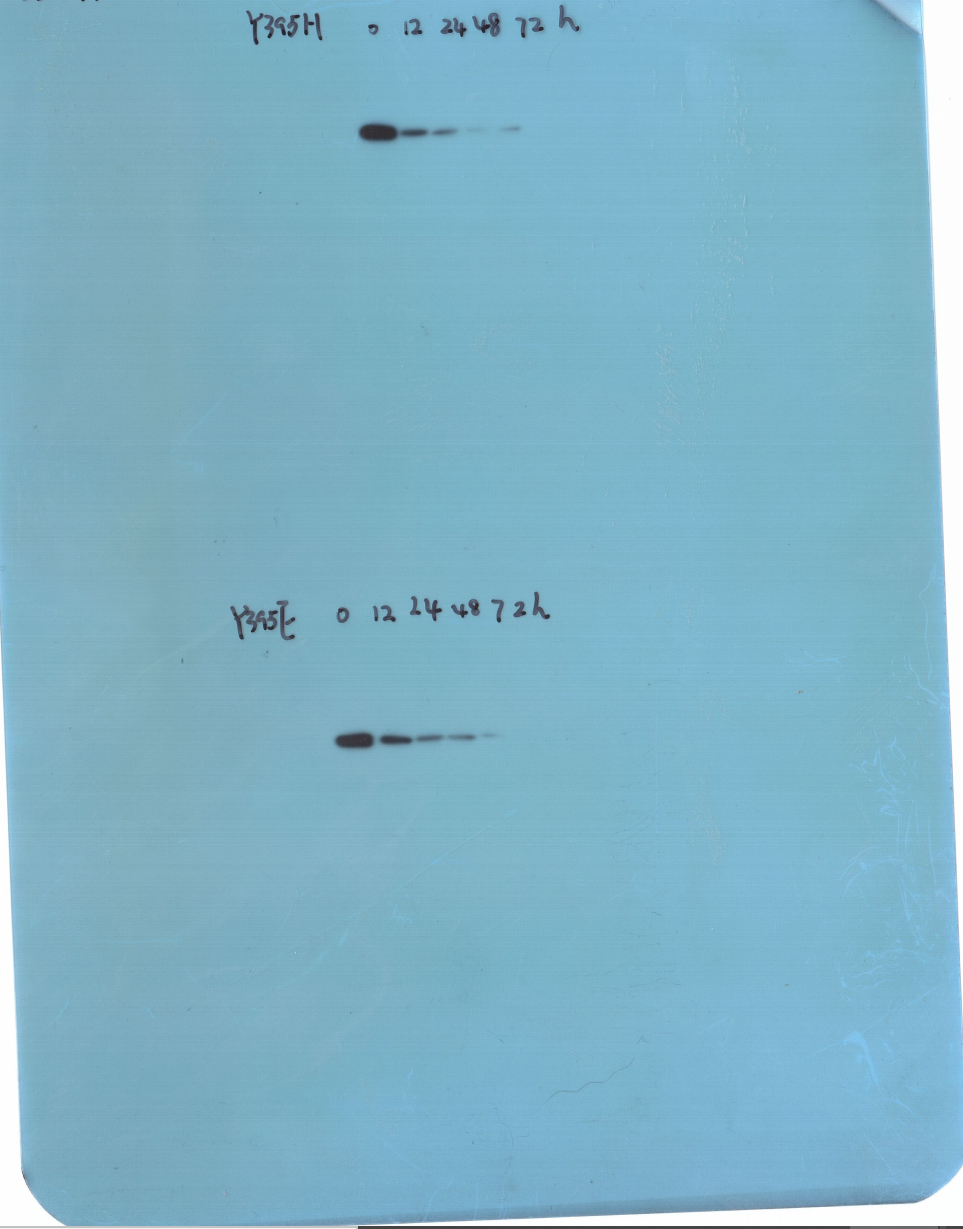

Supplement: Supplementary file 3 [file DataSheet_1.zip › Data sheet/image files of blots/Figure 4B-2.jpg]

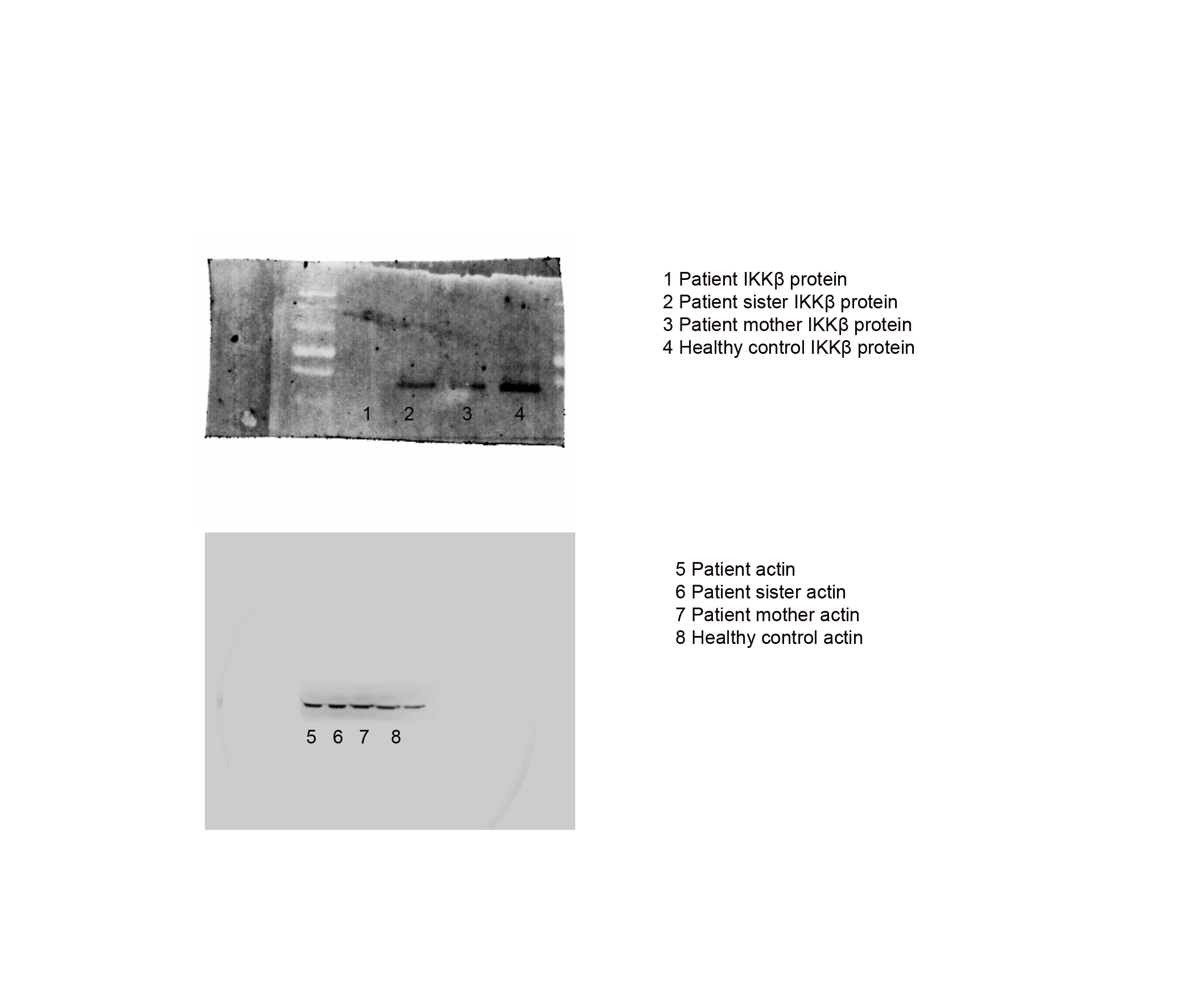

Supplement: Supplementary file 3 [file DataSheet_1.zip › Data sheet/image files of blots/Figure 1E.tif]

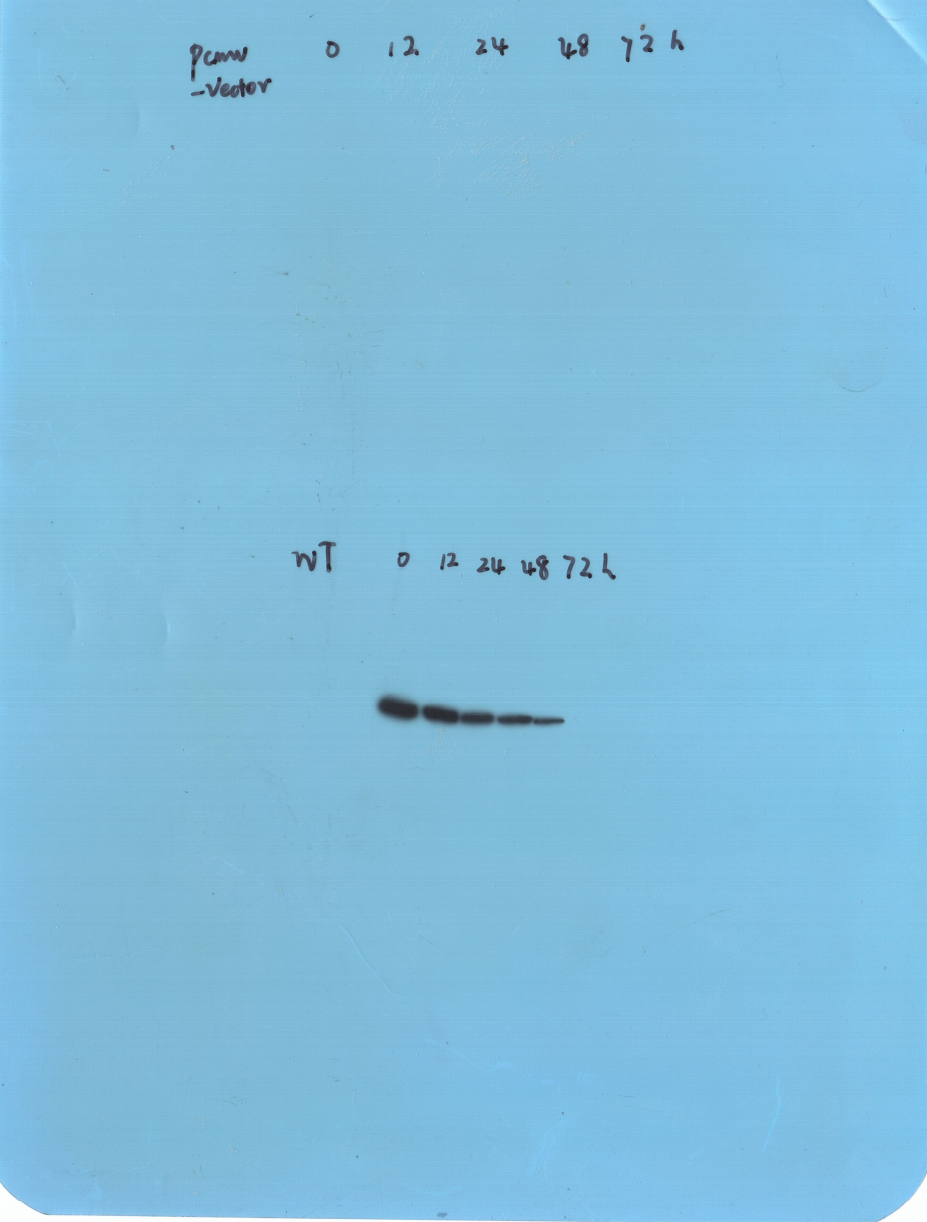

Supplement: Supplementary file 3 [file DataSheet_1.zip › Data sheet/image files of blots/Figure 4B-1.jpg]
