# Supplementary material for: A Novel Homozygous Mutation Destabilizes IKKβ and Leads to Human Combined Immunodeficiency
Source: Front Immunol. 2021 Feb 15;11:517544. doi: 10.3389/fimmu.2020.517544 (PMC7917045; doi:10.3389/fimmu.2020.517544)
Supplement: Supplementary file 4 [file Table_1.docx]

**Supplementary Table 1. Variants in the proband exome**

| Mutation  gene | 1000  Genome | Exon | Nucleotide change | Amino acid change | Gene type | Mutation in normal | Inheritance | Disease |
| --- | --- | --- | --- | --- | --- | --- | --- | --- |
| IKBKB | chr8-  42175238 | 12 | c.1183T>C | p.Y395H | hom | N/A | AR | IKBKB deficiency |
| CD3G | chr11-  118223146 | 6 | c.511T>C | p.Y171H | het | N/A | AR | CD3γ deficiency |
| CFHR5 | chr1-  196952198 | 2 | c.242C>T | p.P81L | het | N/A | AR | Factor H –related protein  deficiencies |
| FOXN1 | chr17-  26861773 | 7 | c.1184C>T | p.P395L | het | N/A | AR | Winged helix  deficiency |
| NCF2 | chr1-  183532445 | 12 | c.936-4C>G | - | het | N/A | AR | XL-CGD |
| RTEL1 | chr20-  62297372 | 7 | c.554A>G | p.Q185R | het | N/A | AR | Dyskeratosis congenita |
| ADA | chr20-  43264927 | 2 | c.36G>A | p.V12V | hom | ADA-  chr20-  43264927 | AR | 1. B- SCID |
| AIRE | chr21-  45717550 | 14 | c.1578T>C | p.D526D | hom | AIRE-  chr21-  45717550 | AR | Autoimmune disease |
| ATM | chr11-  108183167 | 40 | c.5948A>G | p.N1983S | hom | ATM-  chr11-  108183167 | AR | DNA repair-deficiency |
| C1QA | chr1-  22965438 | 3 | c.276A>G | p.G92G | hom | C1QA-  chr1-  22965438 | AR | C1 deficiency |
| C3 | chr19-  6697406 | 21 | c.2745T>C | p.A915A | hom | C3-  chr19-  6697406 | AR,AD | C3 deficiency |
| C3 | chr19-  6702157 | 19 | c.2421G>C | p.V807V | hom | C3-  chr19-  6702157 | AR,AD | C3 deficiency |
| C6 | chr5-  41158863 | 13 | c.1881C>T | p.D627D | hom | C6-  chr5-  41158863 | AR | C6 deficiency |
| C8A | chr1-  57340727 | 3 | c.277C>A | p.Q93K | hom | C8A-  chr1-  57340727 | AR | C8 deficiency |
| C8B | chr1-  57422484 | 3 | c.349G>A | p.G117R | hom | C8B-  chr1-  57422484 | AR | C8 deficiency |
| C8G | chr9-  139840543 | 4 | c.353A>G | p.D118G | hom | C8G-  chr9-  139840543 | AR | C8 deficiency |
| CARD11 | chr7-  2946461 | 25 | c.3276A>G | p.R1092R | hom | CARD11-  chr7-  2946461 | AR,AD | Autoimmune lymphoproliferative syndrome |
| CARD11 | chr7-  2957005 | 20 | c.2622A>G | p.P874P | hom | CARD11-  chr7-  2957005 | AR,AD | Autoimmune lymphoproliferative syndrome |
| CD27 | chr12-  6560473 | 6 | c.698A>G | p.H233R | hom | CD27-  chr12-  6560473 | AR | lymphohistiocytosis syndrome |
| CFHR4 | chr1-  196874356 | 3 | c.375G>T | p.E125D | hom | CFHR4-  chr1-  196874356 | AR | Complement factor H-related protein deficiency |
| CFI | chr4-  110678925 | 7 | c.898A>G | p.T300A | hom | N/A | AR | Complement factor I deficiency |
| -- | chr11 67765163 67765164 | -- | -- | -- | hom | chr11 67765163 67765164 | AR | Herpes simplex encephalitis (HSE) |
| ORAI1 | chr12 122064773 122064780 | 1 | c.127_132del | p.43_44del | hom | chr12 122064773 122064780 | AR | ORAI-I deficiency |
| -- | chr17 26727722 26727723 | -- | -- | -- | hom | chr17 26727722 26727723 | AR | Vitamin B12 and folate metabolism disorders |
| -- | chr8 48805816 48805817 | -- | -- | -- | hom | chr8 48805816 48805817 | AR | T-B- SCID |
| CIITA | chr16-  10995933 | 7 | c.520A>G | p.R174G | hom | CIITA-  chr16-  10995933 | AR | MHC-class II deficiency |
| CIITA | chr16-  11002904 | 10 | c.924G>A | p.T308T | hom | CIITA-  chr16-  11002904 | AR | MHC-class II deficiency |
| CIITA | chr16-  11002927 | 10 | c.947A>G | p.Q316R | hom | CIITA-  chr16-  11002927 | AR | MHC-class II deficiency |
| CIITA | chr16-  11016045 | 15 | c.1419C>T | p.C473C | hom | CIITA-  chr16-  11016045 | AR | MHC-class II deficiency |
| CORO1A | chr16-  30198151 | 4 | c.336A>G | p.P112P | hom | CORO1A-  chr16-  30198151 | AR | T-B＋ SCID |
| CTSC | chr11-  88045583 | 3 | c.458T>C | p.I153T | hom | CTSC-  chr11-  88045583 | AR | Initiative deficiency |
| CYBA | chr16-  88713236 | 4 | c.214T>C | p.Y72H | hom | CYBA-  chr16-  88713236 | AR | Explosive respiratory defects |
| CYBA | chr16-  88717386 | 1 | c.36A>G | p.E12E | hom | CYBA-  chr16-  88717386 | AR | Explosive respiratory defects |
| DCLRE1C | chr10-  14976414 | 6 | c.298C>T | p.L100L | hom | DCLRE1C-chr10-  14976414 | AR | Omenn syndrome |
| DKC1 | chrX-  153994596 | 5 | c.369G>T | p.T123T | hom | DKC1-  chrX-  153994596 | XL | Congenital dyskeratosis |
| DNMT3B | chr20-  31386347 | 12 | c.1284T>C | p.C428C | hom | DNMT3B-  chr20-  31386347 | AR | DNA repair-deficiency |
| DNMT3B | chr20-  31386449 | 12 | c.1386T>C | p.Y462Y | hom | DNMT3B-  chr20-  31386449 | AR | DNA repair-deficiency |
| DNMT3B | chr20-  31387954 | 16 | c.1700-5C>G | -- | hom | DNMT3B-  chr20-  31387954 | AR | DNA repair-deficiency |
| DNMT3B | chr20-  31388636 | 17 | c.1846-5T>C | -- | hom | DNMT3B-  chr20-  31388636 | AR | DNA repair-deficiency |
| DOCK8 | chr9-  286593 | 2 | c.85C>A | p.P29T | hom | DOCK8-  chr9-  286593 | AR | DOCK8 deficiency |
| DOCK8 | chr9-  429719 | 34 | c.4191T>C | p.F1397F | hom | DOCK8-  chr9-  429719 | AR | DOCK8 deficiency |
| DOCK8 | chr9-  433978 | 36 | c.4886+3A>G | -- | hom | DOCK8-  chr9-  433978 | AR | DOCK8 deficiency |
| DOCK8 | chr9-  441952 | 40 | c.5133G>A | p.E1711E | hom | DOCK8-  chr9-  441952 | AR | DOCK8 deficiency |
| FPR1 | chr19-  52249702 | 2 | c.546C>A | p.P182P | hom | FPR1-  chr19-  52249702 | AR | Initiative deficiency |
| FPR1 | chr19-  52250216 | 2 | c.32T>C | p.I11T | hom | FPR1-  chr19-  52250216 | AR | Initiative deficiency |
| GATA2 | chr3-  128205860 | 2 | c.15C>G | p.P5P | hom | GATA2-  chr3-  128205860 | AD | Other deficiency |
| GFI1 | chr1-  92946479 | 4 | c.465C>T | p.C155C | hom | GFI1-  chr1-  92946479 | AD | Neutrophil dysfunction |
| IKBKB | chr8-  42177163 | 14 | c.1571G>A | p.R524Q | hom | IKBKB-  chr8-  42177163 | AR | IKBKB deficiency |
| ISG15 | chr1-  949654 | 2 | c.294A>G | p.V98V | hom | ISG15-  chr1-  949654 | AR | Mendelian susceptibility to mycobacterial disease |
| ITGB2 | chr21-  46311813 | 11 | c.1323T>C | p.V441V | hom | ITGB2-  chr21-  46311813 | AR | Initiative deficiency |
| ITGB2 | chr21-  46314907 | 9 | c.1062A>T | p.Q354H | hom | ITGB2-  chr21-  46314907 | AR | Initiative deficiency |
| KRAS | chr12-  25368462 | 5 | c.483G>A | p.R161R | hom | KRAS-  chr12-  25368462 | -- | Somatic mutation disease |
| MASP1 | chr3-  186954285 | 11 | c.1374T>C | p.P458P | hom | MASP1-  chr3-  186954285 | AR | MASP1 deficiency |
| MASP2 | chr1-  11087524 | 11 | c.1479C>T | p.S493S | hom | MASP2-  chr1-  11087524 | AR | MASP2 deficiency |
| MCM4 | chr8-  48885436 | 13 | c.1948T>A | p.L650M | hom | MCM4-  chr8-  48885436 | AR | DNA repair-deficiency |
| MEFV | chr16-  3297073 | 4 | c.897T>C | p.D299D | hom | MEFV-  chr16-  3297073 | AR | Defects affecting inflammatory complex |
| MEFV | chr16-  3297175 | 4 | c.795A>G | p.Q265Q | hom | MEFV-  chr16-  3297175 | AR | Defects affecting inflammatory complex |
| MEFV | chr16-  3297181 | 4 | c.789G>A | p.E263E | hom | MEFV-  chr16-  3297181 | AR | Defects affecting inflammatory complex |
| MTHFD1 | chr14-  64882380 | 6 | c.401A>G | p.K134R | hom | MTHFD1-  chr14-  64882380 | AR | Vitamin B12 and folate metabolism disorders |
| NCF4 | chr22-  37271882 | 8 | c.815T>C | p.L272P | hom | NCF4-  chr22-  37271882 | AR | Explosive respiratory defects |
| NFKB2 | chr10-  104159196 | 12 | c.1269A>G | p.P423P | hom | NFKB2-  chr10-  104159196 | AD | More than 2 kinds of serum immunoglobulins reduction, with normal or reduced B cells |
| NFKB2 | chr10-  104160434 | 16 | c.1821A>G | p.A607A | hom | NFKB2-  chr10-  104160434 | AD | Serum immunoglobulins reduction, with normal or reduced B cells |
| PLCG2 | chr16-  81819768 | 2 | c.174T>C | p.A58A | hom | PLCG2-  chr16-  81819768 | AD | PLC γ 2-related antibody deficiency and immune dysfunction (PLAID) |
| PLCG2 | chr16-  81929488 | 13 | c.1149C>T | p.D383D | hom | PLCG2-  chr16-  81929488 | AD | PLC γ 2-related antibody deficiency and immune dysfunction (PLAID) |
| PLCG2 | chr16-  81941319 | 16 | c.1497C>T | p.A499A | hom | PLCG2-  chr16-  81941319 | AD | PLC γ 2-related antibody deficiency and immune dysfunction (PLAID) |
| PMS2 | chr7-  6013049 | 15 | c.2570G>C | p.G857A | hom | PMS2-  chr7-  6013049 | AR | DNA repair-deficiency |
| PMS2 | chr7-  6026775 | 11 | c.1621A>G | p.K541E | hom | PMS2-  chr7-  6026775 | AR | DNA repair-deficiency |
| PMS2 | chr7-  6036980 | 7 | c.780C>G | p.S260S | hom | PMS2-  chr7-  6036980 | AR | DNA repair-deficiency |
| PRF1 | chr10-  72358577 | 3 | c.900C>T | p.H300H | hom | PRF1-  chr10-  72358577 | AR | Familial hemophagocytic syndrome (FHL) |
| PRKCD | chr3-  53220215 | 12 | c.1119G>A | p.E373E | hom | PRKCD-  chr3-  53220215 | AR | Autoimmune lymphoproliferative syndrome |
| PRKCD | chr3-  53223927 | 17 | c.1782C>G | p.T594T | hom | PRKCD-  chr3-  53223927 | AR | Autoimmune lymphoproliferative syndrome |
| PRKCD | chr3-  53224002 | 17 | c.1857T>C | p.P619P | hom | PRKCD-  chr3-  53224002 | AR | Autoimmune lymphoproliferative syndrome |
| RAC2 | chr22-  37622815 | 6 | c.477T>C | p.A159A | hom | RAC2-  chr22-  37622815 | AD | Initiative deficiency |
| RAC2 | chr22-  37637653 | 2 | c.81C>G | p.A27A | hom | RAC2-  chr22-  37637653 | AD | T-B- SCID |
| RAG1 | chr11-  36595600 | 2 | c.746A>G | p.H249R | hom | RAG1-  chr11-  36595600 | AR | T-B- SCID |
| RFX5 | chr1-  151316161 | 9 | c.753C>T | p.L251L | hom | RFX5-  chr1-  151316161 | AR | MHC-class II deficiency |
| SERPING1 | chr11-  57381989 | 7 | c.1438G>A | p.V480M | hom | SERPING1-chr11-  57381989 | AD | C1 inhibitor deficiency |
| SLC29A3 | chr10-  73082563 | 2 | c.52A>G | p.R18G | hom | SLC29A3-  chr10-  73082563 | AR | SLC29A3 mutation |
| SLC29A3 | chr10-  73111408 | 4 | c.473C>T | p.S158F | hom | SLC29A3-  chr10-  73111408 | AR | SLC29A3 mutation |
| SLC29A3 | chr10-  73115941 | 5 | c.714T>C | p.T238T | hom | SLC29A3-  chr10-  73115941 | AR | SLC29A3 mutation |
| SLC29A3 | chr10-  73115942 | 5 | c.715G>A | p.V239I | hom | SLC29A3-  chr10-  73115942 | AR | SLC29A3 mutation |
| SLC29A3 | chr10-  73121913 | 6 | c.976A>G | p.I326V | hom | SLC29A3-  chr10-  73121913 | AR | SLC29A3 mutation |
| SLC29A3 | chr10-  73121945 | 6 | c.1008T>C | p.G336G | hom | SLC29A3-  chr10-  73121945 | AR | SLC29A3 mutation |
| SP110 | chr2-  231050715 | 11 | c.1274T>C | p.L425S | hom | SP110-  chr2-  231050715 | AR | Hepatic veno occlusive disease with immunodeficiency (VODI) |
| SP110 | chr2-  231072709 | 8 | c.895G>A | p.G299R | hom | SP110-  chr2-  231072709 | AR | Hepatic veno occlusive disease with immunodeficiency (VODI) |
| SP110 | chr2-  231077725 | 4 | c.334T>C | p.W112R | hom | SP110-  chr2-  231077725 | AR | Hepatic veno occlusive disease with immunodeficiency (VODI) |
| SPINK5 | chr5-  147475386 | 10 | c.800A>G | p.Q267R | hom | SPINK5-  chr5-  147475386 | AR | Comel-Netherton syndrome |
| SPINK5 | chr5-  147477551 | 11 | c.1004C>T | p.A335V | hom | SPINK5-  chr5-  147477551 | AR | Comel-Netherton syndrome |
| SPINK5 | chr5-  147480027 | 13 | c.1103G>A | p.S368N | hom | SPINK5-  chr5-  147480027 | AR | Comel-Netherton syndrome |
| SPINK5 | chr5-  147480112 | 13 | c.1188T>C | p.H396H | hom | SPINK5-  chr5-  147480112 | AR | Comel-Netherton syndrome |
| SPINK5 | chr5-  147480955 | 14 | c.1258A>G | p.K420E | hom | SPINK5-  chr5-  147480955 | AR | Comel-Netherton syndrome |
| SPINK5 | chr5-  147481430 | 15 | c.1389A>G | p.G463G | hom | SPINK5-  chr5-  147481430 | AR | Comel-Netherton syndrome |
| SPINK5 | chr5-  147486677 | 17 | c.1557C>A | p.G519G | hom | SPINK5-  chr5-  147486677 | AR | Comel-Netherton syndrome |
| SPINK5 | chr5-  147488367 | 18 | c.1659C>T | p.V553V | hom | SPINK5-  chr5-  147488367 | AR | Comel-Netherton syndrome |
| SPINK5 | chr5-  147498019 | 23 | c.2132G>A | p.R711Q | hom | SPINK5-  chr5-  147498019 | AR | Comel-Netherton syndrome |
| SPINK5 | chr5-  147499616 | 25 | c.2358C>T | p.L786L | hom | SPINK5-  chr5-  147499616 | AR | Comel-Netherton syndrome |
| SPINK5 | chr5-  147499670 | 25 | c.2412C>T | p.G804G | hom | SPINK5-  chr5-  147499670 | AR | Comel-Netherton syndrome |
| SPINK5 | chr5-  147505116 | 29 | c.2769G>A | p.A923A | hom | SPINK5-  chr5-  147505116 | AR | Comel-Netherton syndrome |
| SPINK5 | chr5-  147510866 | 31 | c.3009T>C | p.G1003G | hom | SPINK5-  chr5-  147510866 | AR | Comel-Netherton syndrome |
| STXBP2 | chr19-  7711221 | 16 | c.1434T>C | p.D478D | hom | STXBP2-  chr19-  7711221 | AR | Familial hemophagocytic syndrome (FHL) |
| STXBP2 | chr19-  7712277 | 18 | c.1567A>G | p.I523V | hom | STXBP2-  chr19-  7712277 | AR | Familial hemophagocytic syndrome (FHL) |
| TAP2 | chr6-  32797773 | 10 | c.1729A>G | p.M577V | hom | N/A | AR | MHC-class I deficiency |
| TCF3 | chr19-  1619339 | 14 | c.1302A>G | p.S434S | hom | TCF3-  chr19-  1619339 | AD | All serum immunoglobulins reduction, with reduced or absent B cells |
| TCN2 | chr22-  31011610 | 6 | c.776G>C | p.R259P | hom | TCN2-  chr22-  31011610 | AR | Vitamin B12 and folate metabolism disorders |
| TNFRSF13B | chr17-  16842912 | 5 | c.831T>C | p.S277S | hom | TNFRSF13B-chr17-  16842912 | AD, AR | More than 2 kinds of serum immunoglobulins reduction, with normal or reduced B cells |
| TNFRSF13B | chr17-  16855878 | 2 | c.81G>A | p.T27T | hom | TNFRSF13B-chr17-  16855878 | AD, AR | More than 2 kinds of serum immunoglobulins reduction, with normal or reduced B cells |
| TTC7A | chr2-  47251469 | 13 | c.550G>C | p.V184L | hom | TTC7A-  chr2-  47251469 | AR | Immune deficiency with multiple intestinal atresia |
| TTC7A | chr2-  47301029 | 19 | c.1482A>G | p.V494V | hom | TTC7A-  chr2-  47301029 | AR | Immune deficiency with multiple intestinal atresia |
| UNC13D | chr17-  73824121 | 32 | c.3198A>G | p.E1066E | hom | UNC13D-  chr17-  73824121 | AR | Familial hemophagocytic syndrome (FHL) |
| UNC13D | chr17-  73827205 | 27 | c.2599A>G | p.K867E | hom | UNC13D-  chr17-  73827205 | AR | Familial hemophagocytic syndrome (FHL) |
| UNC13D | chr17-  73836162 | 11 | c.888G>C | p.P296P | hom | UNC13D-  chr17-  73836162 | AR | Familial hemophagocytic syndrome (FHL) |
| -- | chr11-  67763224 | -- | -- | -- | hom | N/A | AR | Herpes simplex encephalitis (HSE) |
| WIPF1 | chr2-  175436940 | 5 | c.593C>T | p.P198L | hom | WIPF1-  chr2-  175436940 | AR | Congenital thrombocytopenia |
